# The Impact of Diabetes on the Labour Force Participation, Savings and Retirement Income of Workers Aged 45-64 Years in Australia

**DOI:** 10.1371/journal.pone.0116860

**Published:** 2015-02-23

**Authors:** Deborah Schofield, Michelle Cunich, Simon Kelly, Megan E. Passey, Rupendra Shrestha, Emily Callander, Robert Tanton, Lennert Veerman

**Affiliations:** 1 NHMRC Clinical Trials Centre, University of Sydney, Sydney, Australia; 2 National Centre for Social and Economic Modelling, University of Canberra, Canberra, Australia; 3 University Centre for Rural Health–North Coast, School of Public Health, University of Sydney, Sydney, Australia; 4 School of Population Health, University of Queensland, Brisbane, Queensland, Brisbane, Australia; Erasmus University Rotterdam, NETHERLANDS

## Abstract

**Background:**

Diabetes is a debilitating and costly condition. The costs of reduced labour force participation due to diabetes can have severe economic impacts on individuals by reducing their living standards during working and retirement years.

**Methods:**

A purpose-built microsimulation model of Australians aged 45-64 years in 2010, *Health&WealthMOD2030*, was used to estimate the lost savings at age 65 due to premature exit from the labour force because of diabetes. Regression models were used to examine the differences between the projected savings and retirement incomes of people at age 65 for those currently working full or part time with no chronic health condition, full or part time with diabetes, and people not in the labour force due to diabetes.

**Results:**

All Australians aged 45-65 years who are employed full time in 2010 will have accumulated some savings at age 65; whereas only 90.5% of those who are out of the labour force due to diabetes will have done so. By the time they reach age 65, those who retire from the labour force early due to diabetes have a median projected savings of less than $35,000. This is far lower than the median value of total savings for those who remained in the labour force full time with no chronic condition, projected to have $638,000 at age 65.

**Conclusions:**

Not only does premature retirement due to diabetes limit the immediate income available to individuals with this condition, but it also reduces their long-term financial capacity by reducing their accumulated savings and the income these savings could generate in retirement. Policies designed to support the labour force participation of those with diabetes, or interventions to prevent the onset of the disease itself, should be a priority to preserve living standards comparable with others who do not suffer from this condition.

## Introduction

It is widely recognised that people with diabetes have a higher risk of reducing their labour force participation compared to people without this condition [1–6]. The sizeable costs to both individuals and government in terms of lost income, lost taxation revenue, and increased government support payments, have also been quantified and shown to form a significant part of the indirect costs of diabetes [7]. However, the costs of reduced labour force participation due to diabetes can have economic impacts that are endured by patients well beyond working-age as a result of reduced living standards in retirement.

Despite the long-term effects of ill health on living standards in general, most studies on the economic consequences of diabetes have focused on the immediate impact of this condition only i.e. on how diabetes reduces labour force participation and, in turn, the individual’s income at that time [2, 4–6, 8–11]. However, reducing labour force participation or retiring prematurely due to diabetes limits an individual’s ability to save for their retirement and this reducing their living standards for the rest of their lives. This will be particularly important in the future as governments seek ways of managing this severe, debilitating and costly disease; a disease which currently affects an estimated 246 million people globally [1]. This figure is expected to reach 380 million by 2025 [12] due to the rising occurrence of obesity and sedentary lifestyles, as well as the ageing of the population worldwide [10, 13]. The most recent Burden of Disease report has identified diabetes as the second leading cause of burden of disease in men, and the fourth leading cause of burden of disease in women in Australia [14].

This paper examines the impact of early retirement due to diabetes on the level of savings expected at age 65 years for Australians aged 45–64 years in 2010. It also considers the lower income that these reduced savings will generate in retirement. The cohort of 45–64 years (the ‘Baby Boomers’) is considered to be a particularly important age group as this cohort will form an increasingly large proportion of the population, and has a high rate of early retirement. Furthermore, it is also the age when most of people’s lifetime savings are accumulated [15–17]. This paper estimates the amount by which those who exit the work force early due to diabetes have less savings by the time they reach the traditional retirement age in Australia (65 years) and the impact this is likely to have on their living standards in retirement. In doing so, it moves beyond the immediate impact of lost income on people with diabetes and assesses the additional long-term cost of reduced savings.

## Methods

We used an extended version of a previous microsimulation model of health, disability and labour force participation which we developed [18] called *Health&WealthMOD2030*, to assess the impact of diabetes on labour force participation; the personal savings at age 65 of people aged 45–64 years in 2010; and the income that could be generated by these savings. This model was specifically designed to estimate the economic impacts of ill health on the labour force status of Australians aged 45–64 years between 2010 and 2030; information which was previously unavailable.

The base data file of Health&WealthMOD2030 consists of unit record data for people aged 45–64 years from the two Australian Bureau of Statistics (ABS) Surveys of Disability, Ageing and Carers (SDACs) 2003 and 2009 [19, 20]. These nationally representative household survey data contain information on personal characteristics (such as age, sex, family type and state of residence), socioeconomic variables (such as level of education, income and home ownership), labour force status (such as labour force participation, employment restrictions and retirement), and health and disability (such as chronic conditions, health status, type and extent of disability, support and care required) for each person in the household.

Respondents in the SDACs reported what their main and other chronic health conditions were, and their responses were classified using ICD10 codes by the ABS [19, 20]. In this study, respondents were considered to be out of the labour force due to diabetes if they stated they were out of the labour force due to their illness and listed diabetes as their main chronic condition.

The concatenated SDAC data were reweighted to reflect the profile of the 2010 Australian population aged 45–64 years using the reweighting algorithm GREGWT developed by the ABS to reweight their survey data [21]. This reweighting procedure was used to account for the changes in disability and illness, demographics, labour force participation and other features of the population that occurred between the survey years (2003 and 2009) and 2010.

The SDACs included income, savings and wealth data in ranges only. For the purpose of this paper, we derived more detailed information on income and savings from a separate dynamic population microsimulation model called the Australian Population and Policy Simulation Model (APPSIM) [22]. APPSIM was used to generate annual snapshots of the socio-demographic and economic characteristics (such as income and government support payments) of the Australian population. It was developed, and is owned and maintained, by the National Centre for Social and Economic Modelling (NATSEM; http://www.natsem.canberra.edu.au) at the University of Canberra, and is widely used within the Australian Government [22]. Income and savings data from APPSIM were imputed onto the base file (or base population) of Health&WealthMOD2030 using synthetic matching—a process commonly used in microsimulation modelling [23]. Ten variables common to both SDAC and APPSIM strongly related to income were selected as matching variables: labour force status (4 groups: employed full time, employed part time, unemployed, not in the labour force), income unit type (4 groups: married couple with dependents, married couple only, one parent with dependents, one person), income quintile (5 groups: income quintiles 1st-5th), receiving age pension (2 groups: yes or no), receiving disability support pension (2 groups: yes or no), sex (2 groups: male or female), age (4 groups: 45–49 years, 50–54 years, 55–59 years, 60–64 years), number of hours worked per week (5 groups: 1–15 hours, 26–24 hours, 25–34 hours, 35–40 hours, 41-plus hours), highest educational qualification (2 groups: university or non-university) and home ownership (2 groups: yes or no).

### Estimating Total Savings and Income (Annuity) at Age 65

For the purposes of this study, the level of annual retirement income was estimated as the income that could be obtained by converting savings (the value of superannuation, cash deposits, share portfolios and investment properties) into an income stream at age 65 years. (The family home is not an income producing asset; however an investment property is and thus investment properties are included in the calculation of savings). ‘Superannuation’ is a private retirement pension plan and this form of retirement saving is compulsory for almost all employed Australians. Compulsory contributions are made to superannuation funds by a person’s employer and voluntary contributions can be made by employees [24]. To estimate total savings for each person to age 65, we assumed that respondents continued earning at the same level, with an adjustment to increase earnings in line with long-term average earnings growth rates less inflation (i.e. the real earnings growth rate). This rate was estimated as the change in Average Weekly Ordinary Times Earnings (AWOTE) trend data between May 1989 and May 2009 [25], and inflation was measured as the change in the Consumer Price Index (CPI) between June 1989 and June 2009 [26]. The real earnings growth rate was estimated at 1.60 per cent per annum using this calculation. The overall functioning, data sources, outputs and aims of the model are described in detail in Schofield *et al*. [27] and Kelly *et al*. [28].

To estimate the impact savings would have on an individual’s living standard in retirement, we estimated the value of a lifetime annuity that could be purchased if the individual’s savings were invested at age 65 years. Investment in a lifetime annuity provides the individual with a fixed income until death based on the amount invested, life expectancy and gender.

### Defining Leaving the Labour Force Prematurely Because of Diabetes

The SDACs ask respondents about their current labour force status, and if they respond that they are not in the labour force, the reason for this; in particular, whether they had left the labour force due to ill health. The list of health conditions in the SDACs were classified by the ABS using ICD10 codes. People who were identified as being out of the labour force due to ill health and who nominated diabetes (ICD10 Codes: E10-14, E74.8, E83.3) as their main health condition were considered to be out of the labour force due to diabetes in this study (19). Whilst the SDACs cannot be used to identify the type of diabetes of respondents, we note that the sample of diabetics used in this study are most likely to have Type 2 diabetes based on their age profile (Diabetes Australia (2013) estimates that 89% of people aged 40–59 years with diabetes in Australia have Type 2 diabetes)[29].

### Statistical Methods

Multiple linear regression models for the log of the value of total savings at age 65 and total income from the purchase of an annuity by age 65 were used to estimate the differences between the total savings and annuity that would be available for people working full time with no chronic condition, people working part time with no chronic condition, people working full time with diabetes, people working part time with diabetes, and people not in the labour force due to diabetes. Full time work with no chronic condition was used as the reference group. Four types of assets were included in the measure of total savings in this study: cash, shares, superannuation, and investment properties. All values of economic measures are expressed in 2010 Australian dollars and incomes are expressed as an annual value. Multiple regression analysis was undertaken on log-transformed data in order to satisfy the assumptions of the linear regression model, and diagnostic tests confirmed that the assumptions were satisfied. In order to estimate the total savings at age 65 for the entire Australian population aged 45–64 years in 2010, we performed weighted analyses using weights that represented the number of individuals in the Australian population. All analyses were undertaken using SAS V9.2 (SAS Institute Inc., Cary, NC, USA). All statistical tests were two sided with the significance level set at 5%. All results are presented with their 95% confidence intervals.

## Results

We identified 25 104 people living in private accommodation in the concatenated 2003 and 2009 SDACs who were aged 45–64 years. Of these, 46 respondents were out of the labour force due to diabetes and had valid savings responses (total savings equal to, or more than, $0) and thus were considered in the analysis. Once weighted these respondents represented 11 000 individuals in the Australian population in 2010.

A greater percentage of people who are out of the labour force due to diabetes were projected to have accumulated no savings whatsoever, compared to those who are in the labour force (full or part time with no chronic condition or with diabetes). All individuals who are employed full or part time in 2010 will have accumulated some savings at age 65 (in part because of compulsory superannuation savings); whereas only 91% of people who are out of the labour force due to diabetes will have accumulated some savings by age 65 (Table 1).

**Table 1:** Comparison of simulated total savings and income (annuity) at age 65 for people in full or part time employment with no chronic condition and with diabetes, as well as those who are not in the labour force due to diabetes.

			Total savings at age 65 years (cash, super, shares, other properties)					Total income at age 65 years		
	Total population	N	Total population with savings	%	Mean ($)	Sd	Median ($)	Mean ($)	Sd	Median ($)
Employed full time with no chronic condition	1 414 000	**6 606**	1 414 000	100	1 617 000	2 206 000	638 000	90 000	122 000	36 000
Employed part time with no chronic condition	468 000	**2 373**	468 000	100	1 583 000	2 565 000	321 000	83 000	135 000	17 000
Employed full time with diabetes	89 000	**345**	89 000	100	1 316 000	1 648 000	581 000	75 000	95 000	34 000
Employed part time with diabetes	25 000	**105**	25 000	100	1 482 000	2 340 000	336 000	81 000	131 000	19 000
Not in labour force due to diabetes	11 000	**46**	10 000	90.5	346 000	754 000	35 000	19 000	43 000	2 000

Reflecting the greater proportion of people who are out of the labour force due to diabetes and have no savings, this group also has far lower median total savings. When savings at age 65 years are used to purchase a lifetime annuity, there is also a corresponding marked reduction in retirement income available to people out of the labour force due to diabetes compared to those employed full time.

Those who retire from the labour force prematurely due to diabetes have a median value of total savings of $35 000 by the time they are 65 years. This is approximately one-twentieth of the median value of savings for those who remain in the labour force full time and have no chronic condition (an estimated $638 000 of savings at age 65 years). The median annuity available to these groups was $36 000 for those working full time with no chronic health condition, but only $2 000 for those out of the labour force due to their diabetes.

While the value of total savings at age 65 for those employed full time with diabetes is less than those employed full time without a chronic condition ($581 000 and $638 000 respectively), the value of total savings is similar for those employed part time with or without diabetes (approximately $330 000). All these groups have considerably higher total savings at retirement age than those out of the labour force due to diabetes.

Estimates from multiple regression models for total savings and income (annuity) show (in Table 2) that, after adjusting for age, sex and highest level of education, those who are out of the labour force due to diabetes have significantly lower total savings and income (annuity) at age 65 years than those who were working full time with no chronic condition (P<.0001). Those who were out of the labour force due to their diabetes had 95.5% (95%CI: -98.9 to -81.6) less total savings and 95.5% (95%CI: -99.8 to -81.8) less total income at age 65 than those who were working full time and without a chronic condition. Those employed part time with diabetes also had significantly lower total savings and income from savings (annuity) than those employed full time with no chronic condition (P<.0.0372), with a 31.5 percentage point difference. There was no statistically significant difference in the total savings or income at age 65 of people working full time with diabetes compared to those working full time without diabetes.

**Table 2:** Percentage difference of total savings and income (annuity) at age 65 for people working part time with no chronic condition, part time with diabetes, full time with diabetes, and not in the labour force due to diabetes compared to those working full time with no chronic condition.

	Total savings at age 65 years (cash, super, shares, other property)				Total income at age 65 years			
	% difference	p-value	95% CI		% difference	p-value	95% CI	
Employed full time with no chronic condition		*Reference*				*Reference*		
Employed part time with no chronic condition	-35.3	<.0001	-42.5	-27.3	-35.5	<.0001	-42.5	-27.3
Employed full time with diabetes	-10.1	0.2025	-23.7	5.9	-10.1	0.2025	-23.7	5.9
Employed part time with diabetes	-31.5	0.0372	-52.0	-2.2	-31.5	0.0372	-52.0	-2.2
Not in labour force due to diabetes	-95.5	<.0001	-98.9	-81.6	-95.5	<.0001	-98.9	-81.6

## Discussion

Several studies have examined the costs of lost productivity and work absence related to diabetes [1, 4, 9, 11, 30–32]. Recently, Schofield *et al* [7] reported the costs of early retirement due to diabetes, and the subsequent loss of income, lost taxes and increased benefit payments. While the immediate cost of lost income to the individual (and the resultant loss of taxes and increase in benefit payments to government) is significant, premature exits from the labour force also have long-term impacts on the living standards of those with this disease in old age, such as reduced in retirement.

This paper estimated the extent of the long-term disadvantage felt by those who retire early due to diabetes. It went beyond the immediate impact of only lost income and estimated the additional, long-term cost of reduced savings and retirement incomes. It has demonstrated that people who retire from the labour force early due to diabetes are likely to face lasting financial disadvantage compared to those who remain in full time employment and have no chronic condition before age 65. Those out of the labour force due to diabetes accumulate a lower amount of savings by the time they reach the traditional retirement age of 65 and consequently have access to substantially lower incomes in retirement, compared to those who remain in full time employment and have no chronic condition. The median value of total savings held by individuals who retire early due to diabetes by the time they reach the age of 65 is $35 000, which is 96% lower than the value of savings held by those who remained employed full time and without a chronic condition.

The Australian social security system, through the disability support pension and aged pension, provides some support for people who have less savings in retirement on which to live. (Pension rates: $854.30/fortnight for a single person, $1 288 for a couple; Assets limits: $202 000 for a single (homeowner), $286 500 for a couple (homeowner) applying for a full-rate pension, and $771 750 for a single (homeowner), $1.15 million for a couple applying for a part-rate pension; see http://www.humanservices.gov.au/customer/services/centrelink/age-pension.) Although most people with median savings with or without diabetes would both be eligible for a pension, those without diabetes would typically only be eligible to a part-rate pension due to their higher level of savings (Table 1). Access to the disability support pension requires meeting strict medical guidelines which have been further restricted in recent years.

Due to increased life expectancies, the number of years spent in retirement is increasing and thus retirees need to be able to finance a greater number of years that are lived after work. Australia’s heavily subsidised health care system and its social security system may, to some extent, help to protect persons with enduring and debilitating conditions such as diabetes from being completely reliant upon their relatively small stock of wealth and possible deterioration in living standards. However, it is well documented that those who are dependent upon government support payments as a primary source of income often have poorer living standards than people with employment income [33, 34]. Indeed, those who rely solely on the single aged pension are receiving an amount which is below the poverty line, as it is an amount less than 50% of the median equivalence level income in Australia [35]. Thus, people who exit the labour force (and thus can no longer rely on employment income) will often have little savings upon which they can draw to finance their needs, including the costs associated with an illness such as diabetes, and day to day living expenses [36–38].

Two caveats are noted for this study. Firstly, there may be unobservable factors such as respondent preferences [39] associated with both diabetes and retirement decisions/retirement savings. For example, it is possible that respondents whose environment is not conducive to a healthy lifestyle may also have a higher probability of developing diabetes and thus are also less able to work. Conversely, people who develop diabetes may have factors which increase their need to work (e.g. lower savings, dependent children) yet nonetheless be forced to retire early due to their illness.

Secondly, the study does not take into account the lower life expectancy of individuals with diabetes, compared to those without this condition [40, 41], nor the lower life expectancy of people with different types of diabetes or who experience major complications due to diabetes [42].

The notion that the rate of time preference influences both specific chronic diseases (such as diabetes, obesity) and personal savings directly has only recently been proposed in the literature [43, 44] and has yet to be fully tested. There is limited empirical research on the connection between time preference and diabetes. Sloan et al (2009)[45], using panel data on 1,034 adults in the United Sates, found there was no connection between time and risk preference and laboratory measures of HbA1c levels. In relation to obesity, Gray (2011)[46], using a sample of 2000 households representative of the Netherlands population, reports only a small statistically significant effect of time preference on the risk of developing obesity; and Smith et al (2005)[47], using the National Longitudinal Survey of Youth data (NLSY79) reports only “some evidence” of a positive relationship between time preference and Body Mass Index (BMI) for Hispanic and black men and black women. We have been unable to uncover a single study that examines empirically the effect of time preference on both diabetes and retirement income or savings.

This study used an estimate of the value of a lifetime annuity that could be purchased if the individual’s savings were invested at age 65 to assess lifetime savings for those with versus without diabetes. That is, the annuity factor was the same for individuals with and without diabetes. In theory, individuals with diabetes could draw down on assets faster than those without diabetes as a way of compensating for lower savings. However, this would be a risky strategy, as predicting the timing of individual mortality is very uncertain. Future research on the role of life expectancy in determining the savings behaviour of individuals with diabetes would enhance our understanding of the lifetime savings of those with this condition (and its risk factors). As the findings of this study show, not only will premature retirement due to diabetes limit the immediate income available to individuals, but it will also reduce their longer term living standards by reducing their savings. Thus, with an ageing population, there will be increasing pressure on the government to assist the elderly in maintaining living standards in the future if they cannot finance their retirement from their personal accumulated wealth [48]. Those who have been able to maximise their labour force participation are more likely to have higher incomes and better living standards not only when working but also in retirement [49]. Thus, maintaining the labour force participation of those with common chronic conditions such as diabetes by improving their treatment or adapting their working conditions, or preventing the onset of such diseases, should be priorities for policymakers in order to ensure comparable living standards with those who do not suffer from such conditions.
